# High Content Image Based Analysis Identifies Cell Cycle Inhibitors as Regulators of Ebola Virus Infection

**DOI:** 10.3390/v4101865

**Published:** 2012-09-25

**Authors:** Krishna P. Kota, Jacqueline G. Benko, Rajini Mudhasani, Cary Retterer, Julie P. Tran, Sina Bavari, Rekha G. Panchal

**Affiliations:** 1 PerkinElmer, 940 Waltham, MA 02451, USA; Email: krishna.kota@amedd.army.mil; 2 United States Army Medical Research Institute of Infectious Diseases, 1425 Porter st, Fort Detrick, Frederick, MD 21702, USA; Email: jacqueline.g.benko.ctr@us.army.mil (J.G.B.); rajini.mudhasani@amedd.army.mil (R.M.); cary.retterer@us.army.mil (C.R.); julie.tran2@us.army.mil (J.P.T.); sina.bavari@amedd.army.mil (S.B.)

**Keywords:** ebolavirus, cell cycle, high-content imaging, serum starvation, aphidicolin, nocodazole

## Abstract

Viruses modulate a number of host biological responses including the cell cycle to favor their replication. In this study, we developed a high-content imaging (HCI) assay to measure DNA content and identify different phases of the cell cycle. We then investigated the potential effects of cell cycle arrest on Ebola virus (EBOV) infection. Cells arrested in G1 phase by serum starvation or G1/S phase using aphidicolin or G2/M phase using nocodazole showed much reduced EBOV infection compared to the untreated control. Release of cells from serum starvation or aphidicolin block resulted in a time-dependent increase in the percentage of EBOV infected cells. The effect of EBOV infection on cell cycle progression was found to be cell-type dependent. Infection of asynchronous MCF-10A cells with EBOV resulted in a reduced number of cells in G2/M phase with concomitant increase of cells in G1 phase. However, these effects were not observed in HeLa or A549 cells. Together, our studies suggest that EBOV requires actively proliferating cells for efficient replication. Furthermore, multiplexing of HCI based assays to detect viral infection, cell cycle status and other phenotypic changes in a single cell population will provide useful information during screening campaigns using siRNA and small molecule therapeutics.

## 1. Introduction

The filoviruses, marburgviruses and ebolaviruses, are non-segmented single-stranded negative RNA viruses that cause severe hemorrhagic fever with a mortality rate ranging up to 90% in humans [1]. The development of efficacious therapeutics against filoviruses is hampered, in part, by our limited knowledge of the mechanisms of virus/host interactions at the molecular level and in part, by lack of suitable tools and methods to screen large libraries of small molecules or siRNAs. Recently, we reported the development of a high-throughput, high-content image based screening platform to discover novel regulators of EBOV infection [2,3]. Furthermore, these assays can be applied for siRNA screens to identify or validate host factors regulating filovirus infections [4]. The antiviral inhibitors identified from such primary screens can potentially restrict host cell functions that are critical for cellular growth [5]. One of the simplest form of inhibition is restricting cellular proliferation to varying degrees that eventually causes cell death [6]. 

There is growing evidence that a number of different viruses target the host cell cycle to promote their replication [7]. Depending on the virus category (DNA, RNA or retroviruses), some viruses cause arrest of cells in particular phase of cell cycle so that they can replicate in the resting cells, while others induce proliferation of arrested cells [7,8]. For example, negative strand RNA viruses like, influenza A/WSN/33 (H1N1) prevent the entry of cells from G0/G1 to S (synthesis) phase of the cell cycle [9], while positive strand RNA viruses like infectious bronchitis virus (IBV) induce a G2/M (mitotic)‑phase arrest in infected cells [8,10,11]. Thus, by arresting cells in different stages of the cell cycle these viruses provide favorable conditions for their own replication. At the molecular level, the anaphase promoting complex or cyclosome (APC/C), a multi component ubiquitin ligase and a master cell cycle regulator, is targeted by a number of viruses for their replication [11]. Although, there is no direct evidence to suggest that filoviruses modulate the host cell cycle machinery for their replication, gene expression studies in splenocytes obtained from mice infected with EBOV showed significant down regulation of genes associated with the cell cycle regulation pathways [12]. 

Traditionally, cell cycle studies are performed using flow cytometry, wherein DNA dye such as propidium iodide is used to generate characteristic nuclear DNA content profiles. Additional methods to study cell cycle include measuring the expression patterns of proliferating cell nuclear antigen (PCNA, a marker of G1/S progression) and cyclin B1 (a marker for G2/M transition) by using flow cytometry or immunofluorescence [13]. A multiplex assay utilizing a combination of DNA stains 7‑AAD and BrDU, a nucleoside analog that gets incorporated into newly synthesized DNA, can also be used to resolve cell cycle phases using flow cytometry. More recently high-content imaging (HCI) technology is applied to study the effect of therapeutics on cell cycle at the single cell level [14]. The advantage of using HCI is that the during image analysis, several hundreds of cellular features can be extracted at the single cell level [14]. Phenotypic features such as intensity and localization of fluorescently labeled cellular components such as DNA or protein as well as the size, number, texture and shape of subcellular compartments can be extracted to generate information on biological processes such as cell cycle status, cytotoxicity and alternations in cell morphology [15].

In this study, we first optimized a high-content imaging assay to detect population of cells that are forced to accumulate in certain stages of cell cycle following serum starvation or treatment with specific inhibitors of cell cycle. We then investigated the potential effects of cell cycle arrest on EBOV infection. Cells arrested in G1 phase by serum starvation or G1/S or G2/M phase using aphidicolin and nocodazole respectively, and then infected with EBOV showed reduced virus infection. EBOV infection by itself caused increased accumulation of cells in G1 phase, although this effect was found to be cell type dependent. The implications of these findings are discussed.

## 2. Results and Discussion

### 2.1. Image Based Quantitative Analysis to Determine Cell Cycle Phase

**Figure 1:**
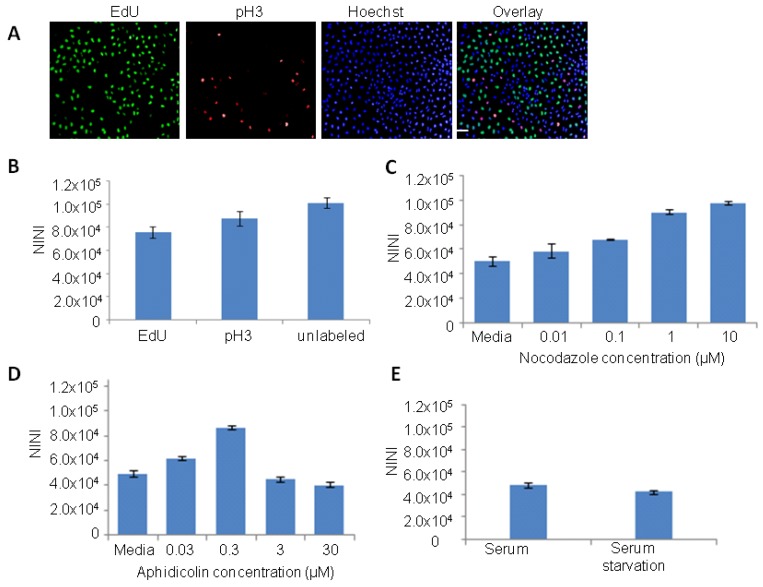
Cell cycle analysis using high-content imaging. (**A**) Representative images of HeLa cells stained with EdU (marker of S phase), pH3 (a marker of M phase) and Hoechst 33342 (marker of DNA). (**B**) Bar graph showing the population of cells in different phases of cell cycle based on normalized integrated nuclear intensity (NINI) derived from the DNA binding dye (Hoechst 33342). NINI value is calculated by measuring the integrated nuclear intensity divided by the number of nuclei within each well. The NINI values derived from mitotic nuclei (pH3 labeled) indicate 4N DNA content, while NINI values for S phase (EdU marked nuclei) range between 0.5 and 1X NINI values of pH3 (2N to 4N DNA content). (**C**,**D**) HeLa cells were treated with indicated concentrations of nocodazole (**C**) or aphidicolin (**D**) for 12 h and then fixed and stained with Hoechst 33342 dye. Cells were imaged and analyzed to extract and calculate the NINI values. (**C**) A dose-dependent increase in NINI values, which corresponds 4N DNA content and a block in G2/M phase was observed with nocodazole. (**D**) Treatment with low aphidicolin concentrations (0.03 and 0.3 μM) results in increasing NINI values which corresponds to a block in G2/M phase, while high aphidicolin concentrations (3 and 30 μM) results in NINI values that correspond to G1 phase. (**E**) HeLa cells were serum starved for 24 h, then fixed and stained with Hoechst 33342 dye. Images were analyzed to extract the NINI values. Serums starved cells showed reduced NINI values compared to control cells incubated with complete medium. Scale bar, 20 µm.

To measure the DNA content and analyze the different phases of the cell cycle by HCI, a multiplex assay was set up. HeLa cells were seeded overnight in 96 well imaging plates, and then treated with EdU (5-ethynyl-2'-deoxyuridine) for 3 h. After fixation the cells were labelled with alexa 488 conjugated small molecule to detect EdU (based on Click-iT^®^ EdU chemisty, Invitrogen) and with antibody against phospho histone 3 (pH3). Cells were subsequently stained with Hoechst 33342 dye, to measure the DNA content based on the nuclear intensity. Cells were defined to be in the S phase by incorporation of the thymidine analog EdU and M phase by immunostaining for the mitotic marker phospho-histone H3 (pH3). Nuclei that were not labelled with EdU or pH3 could be cells that are in G1 or G2 phase. Representative images of cells stained with EdU, Hoechst 33342 and anti pH3 antibody are shown in Figure 1A. During image analysis, normalized integrated nuclear intensity (NINI, (integrated nuclear intensity/total number of nuclei) data derived from Hoechst staining of DNA was collected for all the cells that were imaged. As expected there was an increase in the NINI value in mitotic cells (pH3 labelled cells) that corresponds to 4N DNA content, when compared to cells in S phase or unlabelled cells (Figure 1B). The cells that were not labelled by EdU or pH3 should be in G0/G1 (2N DNA) and G2 (4N DNA) phase. For convenience G0/G1 is simply referred to as G1 phase as they have the same DNA content. Results from these data suggest that if the NINI values for a population of cells treated with compound or siRNA differ significantly from the untreated controls, then it may reflect major changes in cell cycle events in that specific population. 

To further confirm these observations, HeLa cells were treated with known inhibitors that block cells in specific phase of cell cycle and images were analyzed to extract the NINI value. The inhibitor nocodazole is known to inhibit polymerization of microtubules and cause arrest in G2/M phase with 4N DNA content. As expected, our data showed a dose dependent increase in the relative NINI value that corresponds to 4N DNA content compared to untreated control cells (Figure 1C). The compound aphidicolin is known to inhibit DNA polymerase α and can effectively block cells from DNA replication and subsequently arrest cells in the G1/S boundary [17]. HeLa cells treated with low concentrations of aphidicolin (0.03 and 0.3 µM) were arrested in the G2/M phase as measured by an increase in the NINI values. At high aphidicolin concentrations (3 and 30 µM), the NINI values were similar to the untreated control cells, which possibly corresponds to the G1 phase (Figure 1D). Finally, serum starvation of HeLa cells arrested cells in G1 phase, as they showed enrichment of cells with lower NINI value corresponding to 2N DNA content when compared to control cells incubated with medium containing serum (Figure 1E). Based on these observations, we conclude that measurement of the NINI values based on Hoechst 33342 dye staining, can be used to broadly identify cell populations that are accumulating in certain stages of cell cycle compared to control cycling cells. 

### 2.2. Serum Starved Cells Restrict EBOV Infection

**Figure 2:**
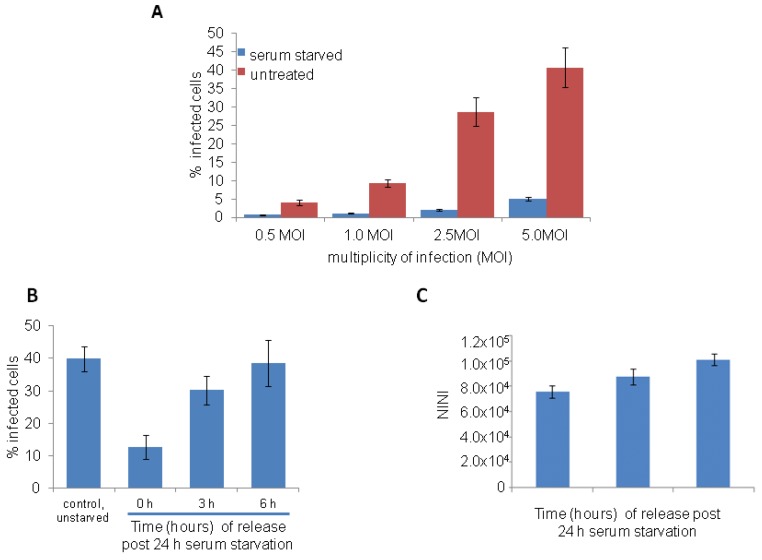
Ebola virus (EBOV) infection is restricted in serum starved HeLa cells. (**A**) HeLa cells were serum starved for 24 h and then infected with different MOI of EBOV for 24 h in complete medium. Cells were stained with anti-GP antibody (6D8) and imaged. Image analysis showed reduced EBOV infection in serum starved cells compared to control cells. (**B**,**C**) HeLa cells were serum starved for 24 h and then infected at 0, 3 h and 6 h following release from the block with 5 MOI of EBOV. After 24 h, cells were fixed, stained with anti-GP antibody (6D8) and Hoechst 33342 dye, and acquired images were analyzed. A time-dependent increase in the EBOV infection (**B**) and increase in NINI values based on Hoechst 33342 dye (**C**) was observed.

To investigate the influence of cell cycle arrest on Ebola virus (EBOV) infection, HeLa cells were serum starved for 24 h and then infected with varying (0.1, 0.5, 2.5 or 5) multiplicity of infection (MOI) of EBOV in medium containing serum. After 1 h virus was removed and cells were incubated for 24 h. To detect EBOV, fixed cells were stained with anti GP antibody to detect the viral glycoprotein (GP) and acquired images were analyzed to calculate the percentage of infected cells. As shown in Figure 2A, serum starved cells showed much reduced percentage of EBOV infected cells compared to cells incubated with complete medium. A reduced EBOV infection was also observed in serum starved A549 (human lung adenocarcinoma cell line) and MCF-10A (human non-transformed mammary epithelial cell line) cells (data not shown). These results suggest that EBOV cannot infect efficiently in cells blocked in G1 phase of the cell cycle. However, if cells are released from the growth arrest by addition of medium containing serum, and infected 3 h or 6 h after the release, a time‑dependant increase in EBOV infection (Figure 2B) and corresponding increase in NINI values (Figure 2C) was observed. Thus, these studies further confirm that EBOV requires actively dividing cells for efficient infection.

### 2.3. Cell Cycle Chemical Inhibitors Regulate EBOV Infection

**Figure 3:**
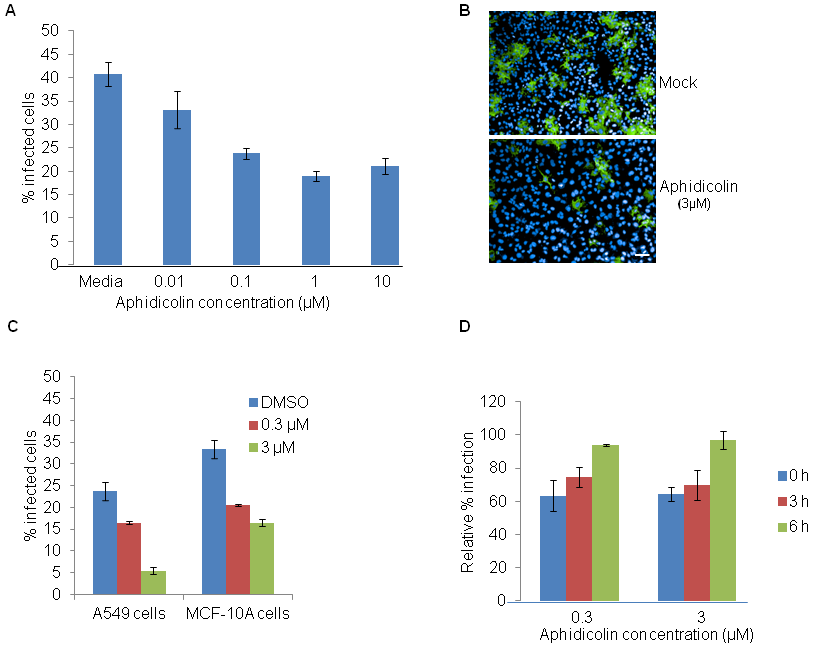
Aphidicolin restricts EBOV infection. (**A**) HeLa cells were pretreated with DMSO control or with indicated concentrations of aphidicolin for 12 h and then infected with 5 MOI of EBOV for 24 h in the absence of the inhibitor. A dose-dependent decrease in percentage of EBOV infected cells was observed. (**B**) Representative images of HeLa cells infected with EBOV and stained with anti-GP antibody (6D8) (green) and nuclear Hoechst dye (blue), following pretreatment with DMSO (top panel) or aphidicolin (3 μM, bottom panel). (**C**) A549 or MCF10A cells were pretreated for 12 h with DMSO control or with 0.3 μM or 3 µM aphidicolin and then infected with 5 MOI of EBOV for 24 h in the absence the inhibitor. A dose-dependent decrease in percentage of EBOV infected cells was observed. (**D**) HeLa cells were released from aphidicolin block and infected with EBOV at 0, 3 or 6 h post-release. Results show a time-dependent increase in efficiency of EBOV infection. Scale bar, 20 µm.

To test the effects of the cell cycle inhibitor aphidicolin on EBOV infection, HeLa cells were treated with different concentrations of aphidicolin for 12 h, washed to remove the inhibitor and then infected with 5 MOI of EBOV. As shown in Figure 3A, a dose dependent decrease in percentage of EBOV infected cells is observed. Representative images of EBOV infected HeLa cells that were pre‑treated with DMSO control or aphidicolin (3 µM) are shown in Figure 3B. Parallel experiments with A549 and MCF-10A cells also showed a dose dependent reduction of EBOV infection (Figure 3C). The efficiency of EBOV infection was then studied in cells that were released from the aphidicolin mediated cell cycle arrest at different time points. HeLa cells were pretreated with aphidicolin for 12 h, washed and replaced with complete medium. At time points of 0, 3 and 6 h, the cells were infected with EBOV for 24 h. A time-dependent increase in EBOV infection was observed (Figure 3D) and found to correlate with normal cell cycle progression (data not shown).

**Figure 4:**
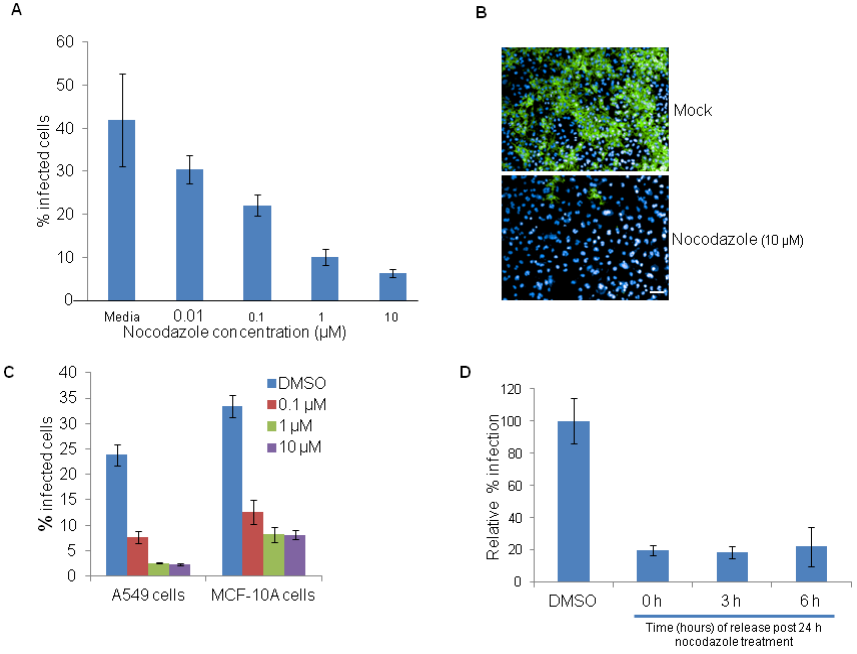
Nocodazole restricts EBOV infection. (**A**) HeLa cells were pretreated for 12 h with DMSO control or with indicated concentrations of nocodazole, and then infected with 5 MOI of EBOV for 24 h in the absence of the inhibitor. A dose dependent decrease in virus infection was observed. (**B**) Representative images of HeLa cells infected with EBOV and stained with anti-GP antibody (6D8) (green) and nuclear Hoechst dye (blue), following pretreatment with DMSO (top panel) or nocodazole (10 µM, bottom panel). (**C**) A549 or MCF-10A cells were pretreated with DMSO control or with 0.1, 1 or 10 µM nocodazole and then infected with 5 MOI of EBOV for 24 h in the absence of the inhibitor. Both A549 and MCF-10A cells showed reduced infection with increasing concentrations of nocodazole. (**D**) HeLa cells were released from nocodazole block and at time points of 0, 3 and 6 h, post release were infected for 24 h with 5 MOI of EBOV. Removal of nocodazole block did not restore the EBOV infection. Scale bar, 20 µm.

Treatment of HeLa cells with different concentrations of nocodazole also showed a dose-dependent reduction in EBOV infection (Figure 4A). Representative images of EBOV infected cells that were pretreated with DMSO control or nocodazole (10 µM) are shown in Figure 4B. Similar, results were observed in nocodazole treated A549 and MCF-10A cells (Figure 4C). However unlike serum starvation or aphidicolin treatments, the release from nocodazole inhibition prior to infection did not result in time-dependent increase of EBOV infection (Figure 4D). One possible reason is that cells have not yet reached the appropriate cell cycle phase for the virus to establish infection. Further characterization such as kinetics of infection and its impact on cell cycle is required to address the mechanism of action. Collectively our data suggests that EBOV infection is favored in actively proliferating cells. 

### 2.4. Modulation of Cell Cycle by EBOV Is Cell Type Dependent

**Figure 5:**
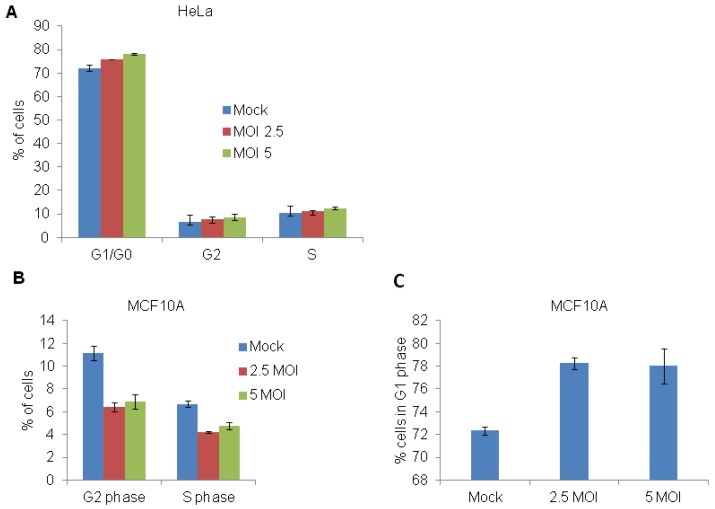
EBOV increases G0/G1 subpopulation in MCF-10A cells but not in HeLa cells. (**A**,**B**) HeLa cells plated in 10 cm dishes were infected with EBOV at 0, 2.5, 5 or 10 MOI for 24 h (**A**) and then subjected to flow cytometry for cell cycle analysis. (**C**) EBOV infection in MCF-10A cells caused an approximately 2 fold reduction in G2/M phase (**B**) and concomitant increase in accumulation of cells in G1 phase (**C**).

To determine whether normal cell cycle progression could be inhibited by EBOV, HeLa cells were infected with 0, 2.5, 5 or 10 MOI of EBOV for 24 h (Figure 5A) and analyzed by flow cytometry following propidium iodide staining. At 24 h, there was no difference in the cell cycle profile between the uninfected and the EBOV infected cells, with majority of the cells accumulated in G0/G1 phase (Figure 5A). At 48 h, there was an increase in cell death with increasing MOI of the virus (data not shown). Cell cycle analysis of EBOV infected A549 cells also did not display any cell cycle defects (data not shown). On the contrary, EBOV infection of MCF-10A cells showed an approximately 2 fold decrease in the percentage of cells in G2/M phase (Figure 5B) and increase accumulation of cells in G1 phase (Figure 5C). The observed difference in EBOV mediated cell cycle modulation between HeLa and MCF10A cells could be due to the nature of cell type. HeLa cells are transformed and aneuploid where as MCF10A are near diploid and untransformed.

## 3. Experimental Section

HeLa, A549 and MCF-10A cells were obtained from ATCC (Rockville, MD) and grown in a 5% CO_2_ humidified incubator at 37 °C. HeLa and A549 cells were cultured in Dulbecco’s Modified Eagle's Medium (DMEM) and F-12K medium (ATCC, cat # 30-2004) respectively containing 10% heat inactivated fetal bovine serum (FBS). The growth media for MCF-10A contains several additives and is composed of DMEM/F12 media supplemented with 5% horse serum, 20 ng/mL epidermal growth factor (EGF, Peprotech), 10 μg/mL insulin (Sigma), 0.5 μg/mL hydrocortisone (Sigma, H-0888) and 100 ng/mL cholera toxin [16]. Medium containing all the required ingredients for growth of the individual cells is referred to in the text as complete medium. For all High-content imaging experiments, HeLa, A549 and MCF-10A cells were seeded at a density of 15,000 cells/well in 96-well imaging plates (Greiner Bio-one, Monroe, NC). For flow cytometry studies, cells were seeded at 1 × 10^6^ cells per 10 cm tissue culture treated plates. 

### 3.1. Serum Starvation

HeLa, MCF-10A or A549 cells were seeded in 96 well imaging plate (15,000 cells/well). Next day cells were either left untreated or washed and incubated with serum free medium for 24 h. To release the cells from the cell cycle block, cells were washed with regular medium containing serum and further incubated in complete medium for the time points indicated in the experiments. All experiments were performed in triplicates and repeated two independent times.

### 3.2. Compound Treatments

Nocodazole (Sigma, M1404) and aphidicolin (Sigma A0781) stock solutions were made in DMSO and diluted to the required concentrations in the respective culture media. For all experiments, cells were incubated with the appropriate concentrations of the inhibitors for 12 h. For the release experiments, compound treated cells were washed and replaced with complete medium. 

### 3.3. Ebola Virus Infection and Staining for High-Content Imaging

For Ebola infection assays, HeLa or A549 or MCF-10A cells seeded in 96-well imaging plates were either left untreated or treated with different conditions (serum starvation or cell cycle inhibitors). After appropriate time points, cells were washed and infected with Ebola virus (EBOV) variant Kitwit at different time points and with different MOI, as indicated in the individual experiments. After 24 h, cells were fixed in 10% formalin for 3 days before processing for immunofluorescence staining. Cells were washed with PBS and then incubated at room temperature (RT) with blocking buffer (3% Bovine serum albumin (BSA)-PBS). For detecting viral GP expression, cells were incubated with anti-GP (6D8) antibody for 1 h at RT. After washing cells were stained for 1 h at RT with goat anti-mouse Alexa Fluor^®^ 488 secondary antibody. The cells were washed with PBS and then stained with the HCS cellmask Deep Red cytoplasmic/nuclear stain (Invitrogen, 5 mg/mL diluted in PBS) and nuclear dye Hoechst 33342 (Invitrogen, 1 mg/mL diluted in PBS). All EBOV infection experiments were performed in triplicates and repeated at least two independent times. 

### 3.4. EdU and pH3 Labeling

For EdU labeling and detection, the Click-iT^®^ EdU Alexa Fluor^®^ 488 Imaging Kit (C10337, Invitrogen, Carlsbad, CA, USA) was used as per manufacturers suggested protocol. Briefly, Hela cells grown to 50% confluence in 96 well imaging plate were incubated with 10 μM EdU for 3 h prior to fixation with 3.7% formaldehyde. Cells were then washed in PBS, permeabilized for 10 min with 0.5% Triton X-100 in PBS and again washed in PBS. To detect EdU, cells were incubated for 30 min with Click-iT^®^ reaction cocktail followed by washing with Click-iT^®^ reaction rinse buffer. Mitotic cells were detected using Phospho histone 3 primary antibody (Cell signaling Technology, Danvers, MA, USA) and Alexa Fluor^®^ 568 Goat Anti-Rabbit secondary antibody (Invitrogen, A-11011). After washing cells were stained with the nuclear dye Hoechst 33342 dye. All labeling experiments were performed in triplicates and repeated two independent times. 

### 3.5. Image Acquisition

Automated image acquisition was performed using an Opera confocal reader (model 5025-Quadruple Excitation High Sensitivity [QEHS], PerkinElmer, Waltham, MA, USA). Images were acquired as 2 exposures using a 10× air objective and a bin fac­tor of 1. The first exposure used the 488-nm and 640-nm lasers to excite the viral and cell body fluorophores, respectively, and was directed to the sample with a 405/488/640-nm triple dichroic. The emission light was split by a 580-nm SP dichroic mirror and collected on separate cameras through 562/40-nm and 690/70-nm band-pass filters. The second exposure used the 405-nm and 561-nm lasers to excite the nuclear and Alexa Fluor^®^ 568 respectively, and was directed to the sample with a 405/561/640-nm triple dichroic. 

### 3.6. Image Analysis

Images were analyzed within the Opera environment using standard Acapella scripts. The algorithm was used to identify objects such as nuclei based on Hoechst dye and cytoplasm based on CellMask™ Deep Red cytoplasmic/nuclear stain. The intensity and subcellular localization of viral infection was determined by Alexa Fluor® 488 fluorescence. For each condition, images from 6 fields/well (~1,700 cells) were acquired with the script calculating the percent positive cells and the mean fluorescence intensities in the cytoplasmic, membrane, or nuclear region (for 488 fluorescence) and nuclear size, nuclear intensities using Hoechst channel. To calculate the normalized integrated nuclear intensity (NINI), total integrated nuclear intensity based on Hoechst channel was divided by the total number of nuclei per well.

### 3.7. Flow Cytometry Analysis

HeLa, A549 and MCF-10A cells were infected with EBOV for 24 or 48 h. Adherent cells were removed with trypsin and suspended in PBS. The recovered cells were then fixed with 70% ice cold ethanol for a minimum of 1 h before being washed in cold PBS. All samples were incubated at 37 °C with 0.5mg/mL RNAse A (Qiagen) for 1 h before the addition of 10 μL of 1mg/mL propidium iodide solution (Sigma). Samples were acquired on a Beckman Coulter Gallios with a stopping gate of 100,000 total events and analyzed with FlowJo software (Treestar). 

## 4. Conclusions

In this study, we report the development of an HCI assay to quantitate different phases of cell cycle. During image analysis, the “integrated nuclear intensity” measurement based on nuclear dye Hoechst 33342 was normalized with cell number data to allow rapid evaluation of cells arrested in different phases of cell cycle. An added advantage of image-based cell cycle analysis is it can distinguish G2 and M phase of the cells, as the latter have smaller nuclei (data not shown). On the contrary flow cytometry analysis of cell cycle which is based on DNA stain alone cannot distinguish cells in G2 and M phase (both have the same 4N DNA content). During HCI based small molecule screening, there is the possibility that the compounds may target multiple pathways and thereby block cells in certain phase of cell cycle. Our future studies will focus on extending our current methods to extract other phenotypic features such as nuclear size, nuclear intensity, morphology and texture and analyze cell cycle at the single cell level using image-based flow cytometry tools.

Application of HCI assay to study the interactions between EBOV and cell cycle revealed that cells blocked in specific phase of cell cycle restrict EBOV infection. Cells blocked in G1 phase by serum starvation or G2/M phase following nocodazole treatment or G1/S phase by aphidicolin all restricted EBOV infection. Previously published reports show that nocodazole could potentially inhibit EBOV at the entry level [21] or during virus egress [22]. Release of cell cycle block induced by serum starvation or aphidicolin resulted in time-dependent increase of EBOV infection. Thus these results suggest that actively proliferating cells are required for EBOV infection. There is also the possibility that other cellular pathways such as mTOR and autophagy that are activated upon serum starvation [24] may potentially modulate EBOV infection. Understanding, the cellular pathways and molecular mechanisms mediating this potent antiviral effect during serum starvation or using known cell cycle inhibitors is crucial for both therapeutic discovery and to understand the basic biology of EBOV infection 

The ability of EBOV to manipulate cell cycle progression was studied in three different cell types. Interestingly, infection of MCF-10A cells caused accumulation of cells in G1 phase and concomitant decrease of cells in G2/M phase. However, EBOV infection of HeLa or A549 cells did not modulate cell cycle progression.

Collectively, our data suggests that EBOV requires actively dividing cells for efficient infection and EBOV may provide a favourable condition for viral production by inducing a G1 phase cell cycle arrest in infected cells in a cell type dependent manner.

## References

[1] Kuhn J.H. (2008). *Filoviruses*. A compendium of 40 years of epidemiological, clinical, and laboratory studies. Arch. Virol. Suppl..

[2] Opsenica I., Burnett J.C., Gussio R., Opsenica D., Todorovic N., Lanteri C.A., Sciotti R.J., Gettayacamin M., Basilico N., Taramelli D. (2011). A chemotype that inhibits three unrelated pathogenic targets: The botulinum neurotoxin serotype A light chain, P. falciparum malaria, and the Ebola filovirus. J. Med. Chem..

[3] Panchal R.G., Reid S.P., Tran J.P., Bergeron A.A., Wells J., Kota K.P., Aman J., Bavari S. (2011). Identification of an antioxidant small-molecule with broad-spectrum antiviral activity. Antivir. Res..

[4] Spurgers K.B., Alefantis T., Peyser B.D., Ruthel G.T., Bergeron A.A., Costantino J.A., Enterlein S., Kota K.P., Boltz R.C., Aman M.J. (2010). Identification of essential filovirion-associated host factors by serial proteomic analysis and RNAi screen. Mol. Cell. Proteomics.

[5] Shum D., Smith J.L., Hirsch A.J., Bhinder B., Radu C., Stein D.A., Nelson J.A., Fruh K., Djaballah H. (2010). High-content assay to identify inhibitors of dengue virus infection. Assay Drug Dev. Technol..

[6] Lyman S.K., Crawley S.C., Gong R., Adamkewicz J.I., McGrath G., Chew J.Y., Choi J., Holst C.R., Goon L.H., Detmer S.A., Vaclavikova J. (2011). High-content, high-throughput analysis of cell cycle perturbations induced by the HSP90 inhibitor XL888. PLoS One.

[7] Davy C., Doorbar J. (2007). G2/M cell cycle arrest in the life cycle of viruses. Virology.

[8] Dove B.K., Bicknell K., Brooks G., Harrison S., Hiscox J.A. (2006). Infectious bronchitis coronavirus induces cell-cycle perturbations. Adv. Exp. Med. Biol..

[9] He Y., Xu K., Keiner B., Zhou J., Czudai V., Li T., Chen Z., Liu J., Klenk H.D., Shu Y.L. (2010). Influenza A virus replication induces cell cycle arrest in G0/G1 phase. J. Virol..

[10] Dove B., Brooks G., Bicknell K., Wurm T., Hiscox J.A. (2006). Cell cycle perturbations induced by infection with the coronavirus infectious bronchitis virus and their effect on virus replication. J. Virol..

[11] Mo M., Shahar S., Fleming S.B., Mercer A.A. (2012). How viruses affect the cell cycle through manipulation of the APC/C. Trends Microbiol..

[12] Panchal R.G., Bradfute S.B., Peyser B.D., Warfield K.L., Ruthel G., Lane D., Kenny T.A., Anderson A.O., Raschke W.C., Bavari S. (2009). Reduced levels of protein tyrosine phosphatase CD45 protect mice from the lethal effects of Ebola virus infection. Cell Host Microbe.

[13] Barton K.M., Levine E.M. (2008). Expression patterns and cell cycle profiles of PCNA, MCM6, cyclin D1, cyclin A2, cyclin B1, and phosphorylated histone H3 in the developing mouse retina. Dev. Dyn..

[14] Low J., Huang S., Blosser W., Dowless M., Burch J., Neubauer B., Stancato L. (2008). High-content imaging characterization of cell cycle therapeutics through *in vitro* and *in vivo* subpopulation analysis. Mol. Cancer Ther..

[15] Carpenter A.E. (2007). Image-based chemical screening. Nat. Chem. Biol..

[16] Debnath J., Muthuswamy S.K., Brugge J.S. (2003). Morphogenesis and oncogenesis of MCF-10A mammary epithelial acini grown in three-dimensional basement membrane cultures. Methods.

[17] Krokan H., Wist E., Krokan R.H. (1981). Aphidicolin inhibits DNA synthesis by DNA polymerase alpha and isolated nuclei by a similar mechanism. Nucleic Acids Res..

[18] Spadari S., Focher F., Sala F., Ciarrocchi G., Koch G., Falaschi A., Pedrali-Noy G. (1985). Control of cell division by aphidicolin without adverse effects upon resting cells. Arzneimittelforschung.

[19] Spadari S., Focher F., Kuenzle C., Corey E.J., Myers A.G., Hardt N., Rebuzzini A., Ciarrocchi G., Pedrali-Noy G. (1985). *In vivo* distribution and activity of aphidicolin on dividing and quiescent cells. Antivir. Res..

[20] Groschel B., Bushman F. (2005). Cell cycle arrest in G2/M promotes early steps of infection by human immunodeficiency virus. J. Virol..

[21] Yonezawa A., Cavrois M., Greene W.C. (2005). Studies of ebola virus glycoprotein-mediated entry and fusion by using pseudotyped human immunodeficiency virus type 1 virions: Involvement of cytoskeletal proteins and enhancement by tumor necrosis factor alpha. J. Virol..

[22] Ruthel G., Demmin G.L., Kallstrom G., Javid M.P., Badie S.S., Will A.B., Nelle T., Schokman R., Nguyen T.L., Carra J.H., Bavari S., Aman M.J. (2005). Association of ebola virus matrix protein VP40 with microtubules. J. Virol..

[23] Pyeon D., Lambert P.F., Ahlquist P. (2005). Production of infectious human papillomavirus independently of viral replication and epithelial cell differentiation. Proc. Natl. Acad. Sci. U. S. A..

[24] Lee Y., Lee H.Y., Gustafsson A.B. (2012). Regulation of Autophagy by Metabolic and Stress Signaling Pathways in the heart. J. Cardiovasc. Pharmacol..

